# Contribution of the BRAF oncogene in the pre-operative phase of thyroid carcinoma

**DOI:** 10.3892/ol.2013.1359

**Published:** 2013-05-22

**Authors:** HOMERO GUSTAVO CORREIA RODRIGUES, ALANA ABRANTES NOGUEIRA DE PONTES, LUIS FERNANDO ADAN

**Affiliations:** 1 School of Medicine, Federal University of Campina Grande, Campina Grande 58.429-140;; 2 School of Medicine, Federal University of Bahia, Salvador, Bahia 40025-101, Brazil

**Keywords:** fine-needle aspiration, BRAF mutation, papillary thyroid carcinoma

## Abstract

Numerous experiments have been conducted over the last few years aiming to identify molecular markers that show the diagnostic accuracy of fine-needle aspiration (FNA), particularly in thyroid lesions that are considered indeterminate. Using certain search arguments and previously defined criteria, 37 studies reporting experiments with the BRAF mutation in pre-operative FNA of the thyroid were selected from the electronic databases PUBMED, MEDLINE, SCOPUS and LILACS, in order to gather evidence with regard to the possible contribution of BRAF in the management of thyroid carcinoma. There were no cases positive for BRAF in follicular carcinomas (FTCs), Hürthle cell carcinomas (HCCs) or medullary thyroid carcinomas (MTCs). Among the 11 cases of anaplastic thyroid carcinomas (ATC), three showed positive results for the BRAF mutation. The number of cases positive for BRAF among the benign lesions was not significant. The average prevalence of BRAF-positive cases in papillary carcinomas (PTC) was 58.6%, while in follicular variants of papillary carcinoma (FVPTC), the average prevalence was 29.6%. For lesions diagnosed as indeterminate or suspicious, the average prevalence of BRAF positivity in PTC was 48.5%. The experiments included in the present study indicated a specificity of almost 100% and a high predominance of the BRAF mutation in PTC, distinguishing the marker in the planning and medical management of papillary carcinoma of the thyroid.

## Introduction

Malignant thyroid alterations are characterized by clinical and pathological variations. They are the most frequent malignant alterations of the endocrine system and the number of cases has progressively increased over the last few years (1). The annual incidence of thyroid nodules clinically detected in adults is estimated at 0.1%, with a prevalence of 4–7% in investigations using palpation, 30–50% in series that use ultrasound and 50% in autopsies (2,3). Fine-needle aspiration (FNA) represents the main pre-operative tool for diagnosing thyroid nodules, due to its technical simplicity and low cost and a reported sensitivity and specificity of 70–98 and 55–100%, respectively (4). However, certain limitations to FNA exist, due to the fact that the material obtained may not be adequate or sufficient, as its volume and quality depend on the technical executor and/or characteristics of the nodule. FNA may also be indeterminate in light of the architectural pattern and the cytological characteristics of the lesion, which may cause misunderstandings, doubts or disagreement, as the diagnosis depends on an interpretation that is frequently based on subtle and subjective criteria (5). Indeterminate situations that do not define whether the lesion is malignant represent 10–20% of the cytopathological diagnoses in material obtained from pre-operative FNA of the thyroid. Often, such limitations mean that patients must undergo surgery and all the inherent risks, not as a therapeutic act, but as a diagnostic method. Consequently, the majority of patients undergo surgery and, during the histopathological exam of the excised piece, more than two-thirds of the nodules are classified as benign, indicating that surgery was unnecessary. This creates high hospital costs and causes eventual morbidities associated with radical surgery of the thyroid (6,7). Several studies have reported that tests for the identification of common somatic genetic alterations in thyroid cancer may be useful for diagnostic clarification in samples obtained from indeterminate or suspicious FNA. The RAF protein, via the BRAF isoform, has been one of the most investigated mutations for the diagnosis of nodular thyroid lesions, in isolation or combined with other oncogenes (RAS and RET/PTC) in cytological material, and has presented encouraging results (8). The most frequent mutation observed in BRAF involves the translocation of thymine for adenine at position T1799A in exon 15, which causes the substitution of the amino acid valine for glutamic acid at position V600E of the protein. The change in the amino acid activates the protein, as it allows constitutive phosphorylation of the adjacent amino acids, conferring oncogenic capacity (9,10). The objective of the present study was to gather the experiments and results obtained in studies of this oncogene as a way to combine and analyze the contribution of BRAF in pre-operative FNA of the thyroid.

## Materials and methods

A broad review of the literature was conducted using the principles of systematic review. The search strategy included using the electronic bibliographical databases PUBMED, MEDLINE, SCOPUS and LILACS between January 2004 and June 2011. The keywords ‘thyroid’ and ‘fine-needle aspiration’ were combined with ‘BRAF’ and ‘molecular marker’. The inclusion criteria were defined as follows: i) The article should have been written in English, French, Italian, Spanish or Portuguese; ii) the main or secondary objective of the study must have been to evaluate the expression of the proto-oncogene BRAF, in isolation or as part of a panel, in material from FNA; iii) the marker should have been submitted for evaluation in samples obtained at the pre-operative phase; and iv) the histopathology of the piece from the surgical resection must have been considered the gold standard of diagnosis. Using these predefined criteria, two of the authors examined the articles that were recovered. The information extracted from the studies included the year of publication, the name of the periodical, the country where the research group was based, the approval register of the experiment conferred by an ethics committee, the distribution of the sample according to gender and age, the number and histological types of the malignant lesions studied in the investigation, the method of analysis of the BRAF gene, the number of cytopathologists involved in the experiment and the identification of other markers when the study involved experiments in panels.

## Results

The 37 experiments included in the present study were published in 21 different periodicals and were conducted by research groups in eight countries; there were 12 studies in the United States, 10 in Korea, nine in Italy, two in China, one in Germany, one in Japan, one in France and one in Portugal. All of the studies were written in English, with the exception of one that was published in French. The majority of the studies (72.9%) made reference to the approval of the experiment by an ethics committee or equivalent research body that the institution the group belonged to. No gender differentiation was made with regard to the participants in 51.3% of the studies. The other participants were identified as 1,209 females and 446 males. The age of the participants was not mentioned in 45.9% of the studies. Among the studies that indicated the age, it was possible to observe that the average age was 46.1 years.

In total, the experiments involved 3,029 thyroid malignant lesions, these included 2,732 papillary carcinomas of the thyroid (PTC), 183 follicular variants of papillary carcinoma (FVPTC), 79 follicular carcinomas (FTC), 19 medullary carcinomas (MTC), 11 anaplastic carcinomas (ATC) and five Hürthle cell carcinomas (HCC; Table I). Polymerase chain reaction (PCR) direct sequencing was the predominantly employed method for analyzing the presence of the BRAF gene in the samples. Several studies (67.5%) did not register the number of cytopathologists involved in the process or analysis of the results. In ten studies, the BRAF gene was submitted for analysis in a panel with other markers, particularly the oncogene RET/PTC (Table II). The majority of the studies (75.6%) included samples of indeterminate or suspicious FNA, with 1,366 lesions studied altogether (Table III).

There were no BRAF-positive cases in the FTCs, HCCs or MTCs. Among the 11 ATC cases, three were positive for the BRAF mutation. The number of cases positive for BRAF among the benign lesions was not significant. The average prevalence of BRAF-positive cases of PTC was 58.6%, while in FVPTC, the average prevalence was 29.6%. For lesions with indeterminate or suspicious diagnoses, the average prevalence of BRAF-positive cases of PTC was 48.5%.

## Discussion

Thyroid nodules are a common condition, but occasionally represent a challenge in the differentiation of benign and malignant lesions. FNA presents with excellent diagnostic precision in the majority of cases; however, a significant percentage of FNA samples are indeterminate, justifying the efforts of several research groups in identifying molecular markers to improve the diagnostic accuracy of FNA of the thyroid. FNAs that indicate thyroid cancer are rarely false-positive (8). In this case, it is possible to conclude that a biomolecular study of the lesion would have little or no importance. However, even such situations justify new approaches, as cytomorphological study of the lesion is not sufficient for the risk stratification and/or proper establishment of medical management measures of the lesion. In this regard, the BRAF mutation has received special attention in the last few years.

Among the four types of thyroid carcinoma, PTC is the most prevalent, responsible for 80–90% of all malignant neoplasms of the thyroid (10,11) and its incidence has been growing rapidly in several areas of the world (12). The samples of the experiments included in the present study revealed an average prevalence of PTC in the order of 96% [(2,732 + 183) / 3,029 × 100]. It is in this type of lesion that there is a more frequent occurrence of the BRAF mutation. It has been reported that the mutation is present in between 28.8 and 69% of PTCs (13). In the present series, an average prevalence of 58.6±20.8% (range, 15–91%) was obtained. PTC is frequently associated with an excellent prognosis and low mortality, but not all patients share such a result (14). This is mainly due to inaccurate information concerning the aggressiveness and level of the tumor in the pre-operative phase (11).

Several studies (11,15–17) have identified the existence of controversy with regard to surgical planning for patients whose cytological aspirations were malignant or indeterminate, and with regard to whether they should undergo partial or total thyroidectomy. In certain cases of lobectomy, the excised nodule is malignant in the histopathological exam, which inevitably requires a second surgery to complete the thyroidectomy, generating additional costs and increasing the possibility of complications and morbidity.

The analysis of the presence of the BRAF mutation in the material obtained from pre-operative FNA is a useful strategy for the reduction of such imprecision and controversies. The specificity with this analysis has been reported at 100% (18), i.e. BRAF mutations are not identified in benign lesions, instead being present only in malignant lesions, particularly PTCs. The presence of the BRAF mutation does not identify malignant lesions totally (low sensibility), although its presence does offer the certainty of the result being a true positive. Moreover, it has been suggested that individuals whose nodules exhibit the BRAF mutation are patients who are more likely to be submitted for total thyroidectomy surgery, independently from the cytological results (4). In addition, in PTC, the BRAF mutation is intimately associated with extra-thyroidean extension, lymph node metastasis and advanced tumor stages (19,20), which are the main clinicopathological risk factors conventionally associated with the increase of recurrence and mortality rates for thyroid cancer (21). Although there are controversies (13,46,47), the conclusions of a meta-analytical study by Lee *et al* (20) revealed the absence of any correlation between the marker and the patients’ ethnicity, age, gender or tumor size. With such qualifications, the detection of the BRAF mutation in pre-operative FNA makes it possible to speed up patient management, while avoiding other less specific diagnostic tests, such as FNA repetition, scintigraphy or freezing intraoperative assessment (22), as well as the decision concerning the extension of surgical resection to prevent the performance of a second surgery (18).

However, it is clear that the frequency of the BRAF mutation does not occur in a uniform manner among the PTC variants. BRAF mutations are more frequent in the high cell variant, followed by the conventional type and then the follicular variant (20). In the specific case of FVPTC in the present study, the average prevalence of the BRAF mutation was 29.6%. This PTC variant deserves special attention as the cytological diagnosis may be difficult due to the super-imposition of morphological characteristics with benign or non-neoplastic lesions (17). The presence of positivity for the BRAF mutation is not a predictive factor of a worst prognosis in FVPTC as it is largely considered in the other PTC variants (22).

A number of the experiments on the BRAF mutation included in the present study were performed in panel form, mainly with other oncogenes (RAS and RET/PTC). The objective of these experiements was to increase the pre-operative FNA diagnostic sensibility, as the BRAF mutation does not occur together with the RAS mutation or the RET-PTC rearrangement, indicating different genetic alterations in the pathogenesis of the papillary carcinoma (9).

In conclusion, considering the association between the BRAF mutation and tumor extension and aggressiveness, we recommend that the establishment of a BRAF mutation detection routine should be analyzed in order to apply this approach in morphologically suspicious or indeterminate FNA, and for pre-operative planning for thyroid cancer.

## Figures and Tables

**Table I.** Studies, analysis method, number of malignant lesions and results of BRAF detection in FNA pre-operative.

First author (ref.)	Year	Analysis method	Number of malignant lesions / BRAF+					
			PTC	FVPTC	FTC	HCC	ATC	MTC
Salvatore *et al* (17)	2004	PCR - direct sequencing/SSCP	47/23	22/3				
Cohen *et al* (22)	2004	PCR direct sequencing and Mutector assay	27/18	27/4	2/0	1/0	1/1	1/0
Hayashida *et al* (13)	2004	PCR - RFLP	21/5					
Xing *et al* (23)	2004	PCR - colorimetric mutation detection method	16/7		5/0	1/0		
Domingues *et al* (24)	2005	PCR - Direct sequencing	11/3		1/0			1/0
Chung *et al* (25)	2006	PCR - Direct sequencing	107/92		3/0		2/1	
Jin *et al* (26)	2006	PCR - Direct sequencing, colorimetric Mutector assay, LightCycler PCR and allele-specific PCR	45/29	13/2				
Rowe *et al* (16)	2006	LightCycler PCR	19/3					
Pizzolanti *et al* (27)	2007	Real-time allele-specific-PCR	14/10	2/1	1/0			
Sapio *et al* (28)	2007	PCR-MASA	6/4		1/0			1/0
Sapio *et al* (18)	2007	PCR-MASA	21/10		5/0			
Kim *et al* (29)	2008	PCR-Pyrosequencing	73/63	2/0	3/0		1/0	1/0
Zatelli *et al* (4)	2009	PCR-Direct sequencing/RFLP	58/41	16/6	7/0		1/1	6/0
Nikiforov *et al* (15)	2009	LightCycler PCR/FMCA	38/18		6/0		2/0	2/0
Moon *et al* (30)	2009	PCR - Direct sequencing	84/42					
Marchetti *et al* (31)	2009	PCR - Direct sequencing	89/59				2/0	
Bentz *et al* (32)	2009	LightCycler PCR/FMCA	24/18	16/6				
Kwak *et al* (21)	2009	PCR - Direct sequencing	339/213					
Xing *et al* (11)	2009	PCR - Colorimetric mutation detection method	149/68	41/5				
Yip *et al* (14)	2009	PCR-FMCA	44/31					
Kim *et al* (33)	2009	PCR - Pyrosequencing	101/88					
Jo *et al* (34)	2009	PCR - Pyrosequencing	40/30					
Hwang *et al* (12)	2010	PCR - Direct sequencing and allele-specific PCR	135/106					
Lin *et al* (35)	2010	PCR - Direct sequencing	61/21					
Dujardin *et al* (36)	2010	PCR - Direct sequencing	10/7					
Girlando *et al* (37)	2010	PCR - Direct sequencing	44/34	16/9				
Musholt *et al* (38)	2010	PCR - MASA	22/9		4/0		1/0	1/0
Kim *et al* (39)	2010	DPO-based multiplex PCR	263/221		4/0			1/0
Guo *et al* (40)	2010	PCR - Direct sequencing	8/4					
Kwak *et al* (41)	2010	DPO-Based Multiplex PCR	107/86	2/1				
Ohori *et al* (42)	2010	LightCycler PCR	20/3					
Moses *et al* (43)	2010	PCR - Direct sequencing	70/20	19/3	8/0	2/0	1/0	1/0
Cantara *et al* (8)	2010	PCR - Direct sequencing	74/33		3/0	1/0		
Kim *et al* (44)	2011	PCR- Pyrosequencing	169/154		4/0			
Yeo *et al* (45)	2011	PCR- Pyrosequencing	175/95	7/4	6/0			4/0
Adeniran *et al* (46)	2011	PCR - Direct sequencing/SSCP	60/40					
Pelizzo *et al* (47)	2011	PCR - Direct sequencing/MASA	141/98		16/0			

FNA, fine-needle aspiration; PTC, papillary thyroid carcinoma; FVPTC, follicular variant of papillary thyroid carcinoma; FTC, follicular thyroid carcinoma; HCC, Hürthle cell carcinoma; ATC, anaplastic thyroid carcinoma; MTC, medullary thyroid carcinoma; PCR, polymerase chain reaction; MASA, mutant allele-specific amplification; DPO, dual-priming oligonucleotide; RFLP, restriction fragment length polymorphism; SSCP, single-strand conformational polymorphism; FMCA, fluorescence melting curve analysis.

**Table II.** Distribution of studies according to the types and quantity of markers used in panel with the BRAF gene.

First author (ref.)	Year	Markers used
Cantara *et al* (8)	2010	BRAF, RET, RAS, TRK^a^, PAX8^a^
Salvatore *et al* (17)	2004	BRAF, RET
Moses *et al* (43)	2010	BRAF, RET, RAS
Nikiforov *et al* (15)	2009	BRAF, RET, RAS, PAX8^b^
Musholt *et al* (38)	2010	BRAF, RET
Sapio *et al* (18)	2007	GAL-3, BRAF
Sapio *et al* (28)	2007	BRAF, RET, TRK^a^
Pizzolanti *et al* (27)	2007	BRAF, RET
Domingues *et al* (24)	2004	BRAF, RET
Ohori *et al* (42)	2010	BRAF, RET, RAS, PAX8^b^

a No mutation in the samples selected.

b Only one mutation present in the sample.

**Table III.** Distribution of studies according to number of thyroid papillary carcinomas in the indeterminate or suspicious cytological samples and the number positive for BRAF mutation.

First author (ref.)	Total of FNAs	Indeterminate or suspicious lesions / PTC number	Indeterminate or suspicious lesions / BRAF^+^
Moses *et al* (43)	196	137/33 (19 FVPTC)	137/13 (3FVPTC)
Cantara *et al* (8)	235	95/53	95/23
Nikiforov *et al* (15)	86	52/17	52/7
Sapio *et al* (18)	144	94/2	94/10
Rowe *et al* (16)	24	19/19	19/3
Salvatore *et al* (17)	96	34/15 (6 FVPTC)	34/4 (1 FVPTC)
Xing *et al* (11)	45	25/7	25/2
Cohen *et al* (22)	91	55/29 (21 FVPTC)	55/5 (2 FVPTC)
Musholt *et al* (38)	93	19/4	19/1
Dujardin *et al* (36)	25	13/7	13/4
Kim *et al* (39)	279	80/70	80/50
Kwak *et al* (41)	130	30/20	30/16
Ohori *et al* (42)	117	117/20	117/3
Moon *et al* (30)	91	91/84	91/42
Marchetti *et al* (31)	111	52/33	33/18
Bentz *et al* (33)	45	17/17	17/3
Jo *et al* (34)	101	24/9	24/7
Pizzolanti *et al* (27)	156	19/3 (1 FVPTC)	19/2 (1FVPTC)
Sapio *et al* (28)	132	37/6	37/4
Chung *et al* (25)	137	25/5	25/3
Domingues *et al* (24)	24	10/1	10/0
Hayashida *et al* (13)	21	1/1	1/1
Yeo *et al* (45)	209	63/49 (5 FVPTC)	63/14 (3 FVPTC)
Adeniran *et al* (46)	72	34/22	34/10
Pelizzo *et al* (47)	270	164/45	164/30
Jin *et al* (26)	71	12/(^a^)	12/1
Girlando *et al* (37)	91	20/14	20/10
Kim *et al* (29)	103	27/18 (2 FVPTC)	27/13

a Not determined. FNA, fine-needle aspiration; PTC, papillary thyroid carcinoma; FVPTC, follicular variant of papillary thyroid carcinoma.
